# Dynamic changes of genomic methylation profiles at different growth stages in Chinese Tan sheep

**DOI:** 10.1186/s40104-021-00632-9

**Published:** 2021-11-02

**Authors:** Yufang Liu, Qiao Xu, Xiaolong Kang, Kejun Wang, Jve Wang, Dengzhen Feng, Ying Bai, Meiying Fang

**Affiliations:** 1 Department of Animal Genetics and Breeding, National Engineering Laboratory for Animal Breeding, MOA Laboratory of Animal Genetics and Breeding, College of Animal Science and Technology, China Agricultural University, No. 2 Yuanmingyuan West Rd, Beijing, 100193 People’s Republic of China; 2 College of Life Sciences and Food Engineering, Hebei University of Engineering, Handan, 056021 People’s Republic of China; 3 Biotechnology Institute, Nanchang Normal University, Nanchang, 330029 People’s Republic of China; 4 College of Agriculture, Ningxia University, Yinchuan, 750021 People’s Republic of China; 5 College of Animal Science and Veterinary Medicine, Henan Agricultural University, Zhengzhou, 450002 People’s Republic of China; 6 Beijing Key Laboratory for Animal Genetic Improvement, Beijing, 100193 People’s Republic of China

**Keywords:** Chinese tan sheep, Different growth stages, DNA methylation, Whole genome bisulfite sequencing

## Abstract

**Background:**

Tan sheep, an important local sheep breed in China, is famous for their fur quality. One-month-old Tan sheep have white, curly hair with beautiful flower spikes, commonly known as “nine bends”, which has high economic value. However, the “nine bends” characteristic gradually disappears with age; consequently, the economic value of the Tan sheep decreases. Age-related changes in DNA methylation have been reported and may be responsible for age-induced changes in gene expression. Until now, no genome-wide surveys have been conducted to identify potential DNA methylation sites involved in different sheep growth stages. In this study we investigated the dynamic changes of genome-wide DNA methylation profiles in Tan sheep using DNA from skin and deep whole-genome bisulfite sequencing, and compared the DNA methylation levels at three different growth stages: 1, 24, and 48 months old (mon1, mon24, and mon48, respectively).

**Results:**

In this study, 11 skin samples from three growth stages (four for mon1, four for mon24, and three for mon48) were used for DNA methylation analysis and gene expression profiling. There were 52, 288 and 236 differentially methylated genes (DMGs) identified between mon1 and mon24, mon1 and mon48, and mon24 and mon48, respectively. Of the differentially methylated regions, 1.11%, 7.61%, and 7.65% were in the promoter in mon1 vs. mon24, mon24 vs. mon48, and mon1 vs. mon48, respectively. DMGs were enriched in the MAPK and WNT signaling pathways, which are related to age growth and hair follicle morphogenesis processes. There were 51 DMGs associated with age growth and curly fleece formation. Four DMGs between mon1 and mon48 (*KRT71*, *CD44*, *ROR2* and *ZDHHC13*) were further validated by bisulfite sequencing.

**Conclusions:**

This study revealed dynamic changes in the genomic methylation profiles of mon1, mon24, and mon48 sheep, and the percentages of methylated cytosines were 3.38%, 2.85% and 4.17%, respectively. Of the DMGs, *KRT71* and *CD44* were highly methylated in mon1, and *ROR2* and *ZDHHC13* were highly methylated in mon48. These findings provide foundational information that may be used to develop strategies for potentially retaining the lamb fur and thus improving the economic value of Tan sheep.

**Supplementary Information:**

The online version contains supplementary material available at 10.1186/s40104-021-00632-9.

## Background

Varied body structure and organ function is an unavoidable and complex process associated with aging, and results in increased morbidity and mortality [1]. Changes in DNA methylation profiles have been considered a cause of senescence since the Vaniushin group first reported an age-related decrease in methylcytosine levels in the somatic tissues of salmon [2]. Age-related changes in DNA methylation have been demonstrated in mammals, but the generality of this phenomenon in vertebrates remains unclear [3, 4]. In a previous study, CpG-rich areas of the genome became hypermethylated in rhesus monkeys, and there was typically decreased gene expression near transcription start sites [5]. In the study of the relationship between fleece development and age, diverse whole-genome methylation profiles have been found to characterize the two periods of hair follicle (HF) growth (anagen and telogen), which indicates that growth stages affect changes in methylation expression patterns, which in turn affects phenotype [6].

DNA methylation is an important epigenetic mechanism that is involved in regulation of various biological processes [7]. Methylation in the 5′- flanking region of a gene plays a pivotal role in regulating expression [8], and epigenetic markers silence exogenous transposons and imprint genes [9]. This mechanism may produce diverse phenotypes that are capable of responding to environmental changes during maturation [10]. A standard approach to measuring DNA methylation is bisulfite sequencing (BS-seq). BS-seq couples bisulfite conversion of DNA with next-generation sequencing to profile genome-wide DNA methylation at single base resolution [11, 12]. BS-seq was first used in *Arabidopsis thaliana* DNA methylome studies; since then, it has provided accurate, unbiased, and high-coverage DNA methylation landscapes in model animals such as humans, mice, and rats [9, 13–15]. In recent studies, methylation analysis in sheep focused on the methylation levels of both global and the specific genes. For example, utero-specific DNA methylation analysis showed that high DNA methylation in ewes could affect their offspring’s resistance to diseases [16]. In a specific genes study, highly expressed DNA methyltransferase 1 prevented the epithelial progenitor cells in the hair fleece bulb from over-proliferating. Over-proliferating drives differentiation; thus, high expression of DNA methyltransferase 1 can maintain normal hair fleece structure [17].

The Chinese Tan sheep, which is indigenous to China, is a short-tailed breed that provides an important source of high-quality fur. The lustrous curly fleece appears when Tan lambs are 1 month old and is characterized by a natural white color [18]. With aging, their fur quality decreases, which in turn affects their economic value [18, 19]. Moreover, we observed that age also impacts on fur quality of Tan sheep. Previous studies on Chinese Tan sheep mainly focused on genetic evaluation and phenotypes characteristic of fur [20]; very few studies have focused on deciphering the relationship between genome and phenotype at the molecular level, including genomic methylation levels, in Tan sheep at different ages. In previous studies, we assessed the transcriptomic and miRNA differences in skin tissues derived from Tan sheep with different degrees of curly fleece that were in different growth stages (1 and 48 months old) [7, 8]. Genome sequence analysis showed that no mutations occurred in candidate gene sequences at different stages. However, little is known about the biological process of DNA methylation regulation involved in different sheep growth stages.

In this study, we hypothesized that the dynamic changes of DNA methylation modulate the epigenetic changes of different growth stages in Tan sheep. Therefore, the objectives of this study were to determine differentially methylated regions (DMRs) in skin tissues from 1-, 24-, and 48-month-old Tan sheep and to evaluate the correlation between transcriptomic profiles and DNA methylation signatures. These results will provide new insights into the biological mechanisms underlying variation of the growth process and by providing foundational information that could be used to develop strategies for potentially retaining the lamb coat and thus improve the economic value of Chinese Tan sheep.

## Materials and methods

### Animals and sample preparation

**Fig. 1:**
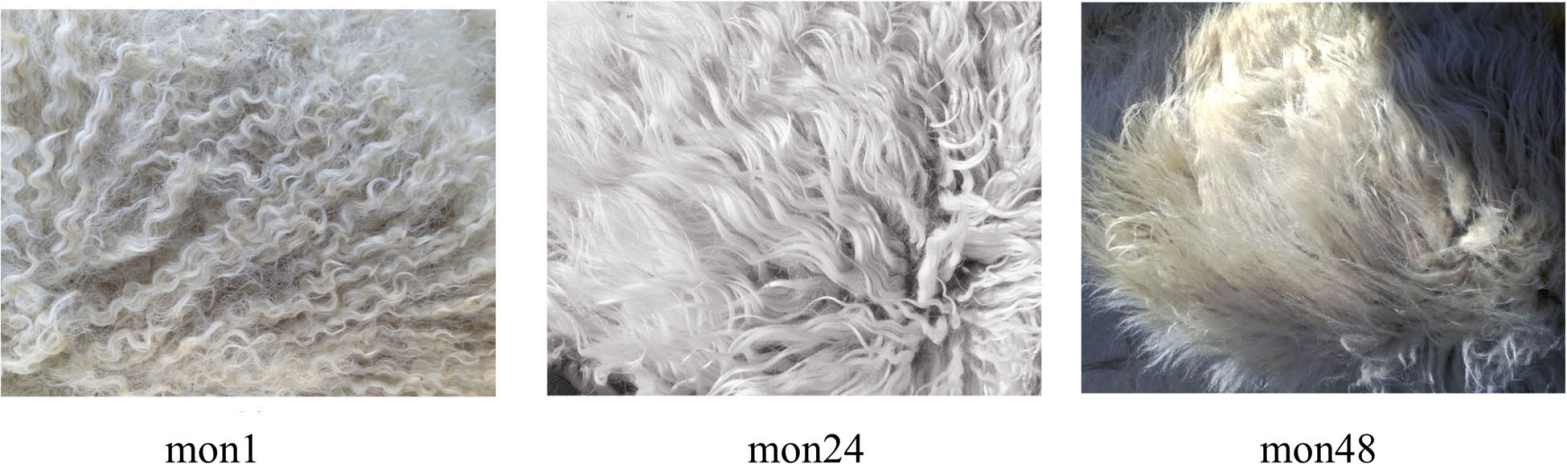
Wool phenotypes in Chinese Tan sheep at three growth stages (mon1, mon24, and mon48 groups)

Eleven female Chinese Tan sheep were used in this study. We sampled skin tissues from the shoulder of differently aged Tan sheep (Fig. 1) for DNA. Animals were divided into three age-based groups that represented different growth stages: 1 month (*n* = 4; mon1 group), 24 months (*n* = 4; mon24 group), and 48 months (*n* = 3; mon48 group). Animals were maintained under identical feeding conditions to minimize external factors. When they were 1, 24, and 48 months old, we cleaned their scapular region, from which the skin tissues were collected using sterilized scalpel blades; samples were immediately frozen in liquid nitrogen and stored at − 80 °C. All resulting wounds were treated with Yunnan Baiyao powder (Yunnan Baiyao Group Co., Ltd., China) to stop the bleeding. By following the manufacturer’s protocol, genomic DNA was isolated using a TIANamp Genomic DNA Kit (TIANGEN, Beijing, China). DNA integrity and quality were evaluated by agarose gel electrophoresis and spectrophotometry with a Nano-Drop spectrophotometer.

### DNA bisulfite treatment and sequencing

After DNA samples were extracted and assessed for quality, bisulfite conversion was performed using the EZ DNA Methylation Direct Kit (Zymo Research Corporation, Irvine, CA, United States). A Covaris S220 sonicator (Covaris, Woburn, MA, United States) was used to fragment genomic DNA to a mean size of approximately 150 bp. After fragmentation, DNA fragments with recessed 3′-ends were repaired using dA to make the ends blunt. Adapters were added according to the manufacturer’s instructions. Following the protocol described in [10], the adapter-modified DNA was then reacted with sodium bisulfate. Bisulfite-treated DNA was PCR amplified, then subjected to paired-end sequencing using the Illumina HiSeq 2500 high-throughput sequencing system (Illumina, San Diego, CA, United States). Reads were 150 nucleotides in length.

### BS-seq read mapping and methyl-cytosine analysis

Reads generated by BS-seq were mapped to the sheep reference genome (Oar_v3.1) from the Livestock Genomics website (http://www.livestockgenomics.csiro.au/) using Bismark (v0.16.3) [14]. In the BS-seq procedure, unmethylated cytosines in genomic DNA were converted into thymines after bisulfite treatment and PCR amplification, but methylated cytosines remained unchanged. The bisulfite conversion rate was calculated using Bismark [14] as the percentage of methylated reads in the total number of clean reads. Only one mismatch was permitted in the “seed” (the high-quality end of the read, default seed length was 28 bases) during alignment. Otherwise, default parameters were used.

The “bismark_methylation_extractor” script packaged with Bismark was used to extract methylation calls. The methylation status of each cytosine with ≥ 5-fold coverage was analyzed using the binomial distribution model. At each position, we used to read depth to test whether the number of detected cytosines exceeded the number expected because of sequencing error. The *P*-value was adjusted by the Benjamini-Hochberg method, and methylated cytosine residues were defined as those with a false-discovery rate (FDR) < 0.05. Next, we estimated the level of differential methylation in pairwise comparisons between the three groups (mon1 vs. mon24, mon1 vs. mon48, and mon24 vs. mon48). This analysis generated coverage statistics for methylated cytosine reads and their contexts (CG, CHG and CHH, where H can be A, T, or C, respectively). Because the methylation of a single C site cannot be discriminated by Bismark, we used the binomial distribution to confirm methylation at each C by examining sites with ≥ 5-fold coverage and FDR < 0.05. The methylation level at a C site was determined using the procedure described in Cokus et al. [21].

To identify differentially methylated CpG sites that are likely to function in gene regulation, we compared mapped CG-containing reads from mon1, mon24, and mon48 sheep. Sites were only considered if they were represented by at least 10 reads in each sheep. A site was classified as differentially methylated if the mon1, mon24, and mon48 versions of the site exhibited different methylation levels. More stringent criteria were then applied to identify methylation level differences [larger than 10% (two-tailed Fisher’s exact test, *P* < 0.05) with an FDR < 0.00005].

### DMR identification and analysis

After removing low-quality reads, all cytosine sites with ≥ 10-fold coverage were aligned to the Ensembl sheep reference genome (Oar v3.1/2.62) using methylKit (v.0.9.2) and eDMR (v0.5.1) [22]. This algorithm identifies DMRs using the bimodal normal distribution of distances between cytosines to optimize the defined region. At least one differentially methylated cytosine and three CpGs were required per DMR, and the difference in methylation levels was required to be greater than 0.2 (0.3 for the CG context) with a Fisher’s exact test *P* < 0.05. The methylation level in a region was calculated as described by Cokus et al. [21].

### Gene ontology (GO) and Kyoto encyclopedia of genes and genomes (KEGG) analyses of differentially methylated genes (DMGs)

DMGs were annotated using the GO and KEGG functional databases to infer gene function. GO enrichment analysis was conducted using the Wallenius non-central hyper-geometric distribution in the GOseq R package [14]. The KEGG database was used to test differentially expressed genes for statistically significant enrichment in biochemical pathways [15]. Interaction networks for selected DMGs were analyzed using the String database (http://string-db.org/) [13].

### DMR validation using BS-seq

Because of the extreme phenotypes of 1- and 48-month-old Tan sheep, this study mainly focused on differences between the mon1 and mon48 groups. DNA was extracted from the skin tissues of seven female Tan sheep (four 1-month-old lambs and three 48-month-old adults). DNA was extracted from frozen skin tissues using extraction kits (Tiangen, Beijing, China). The extracted DNA (concentration: 50 ng/μL) was treated with the EpiTect Bisulfite Kit (QIAGEN, Valencia, CA, USA) according to the manufacturer’s protocol. The DNA samples were treated with bisulfite prior to a PCR, then subjected to pyrosequencing; the primers and conditions are listed in Table 1. The 5′-end of one primer from each pair was conjugated to biotin to allow binding of one DNA strand to streptavidin beads. The 50-μL PCR mixture contained 10 μL of PCR mix buffer (QIAGEN, Valencia, CA, USA), 1 pmol biotinylated forward primer, 1 pmol reverse primer, 2 μL bisulfite-treated genomic DNA, and water.

**Table 1:** Bisulfite-converted DNA PCR primers

Primers name	Primer sequence (5′ to 3′)
*KRT71*-F	GTATAGTGGGGAAGAGGTTTTTATAG
*KRT71*-R	CCACACAAAATCACACAACTAATCAA
*KRT71*-S	AATCACACAACTAATCAAC
*CD44*-F	AATGATGGATGTAGAATAATTTAATTGTTT
*CD44*-R	ACAACCTCAATCTTAAAAATACTATAAC
*CD44*-S	ATTTTATAGATTTGAATATAATTTG
*ROR2*-F	GGGGTTATAAAATAGATGGATATGATTTAG
*ROR2*-R	TTTTTAACAACTCCTCCTTTATCTCT
*ROR2*-S	AGTAGTTTTTATAATTTTGTTTGAG
*ZDHHC13*-F	AGGTTGGTGTAAAAGTAATTGT
*ZDHHC13*-R	CATAACCACATTTATTTAAAAATATATTCC
*ZDHHC13*-S	TGTGGTTTTGTATTGTTGA

*Note*: F represents forward primers; R represents reverse primers; S represents sequencing primers

The degree of methylation was expressed as the proportion of 5-methylated cytosines (mC). Non-CpG cytosine residues were used as built-in controls to verify bisulfite conversion. Methylation measurements were standardized by processing batch number using a mean value of 0 and standard deviation of 1. The methylated DNA assays were designed to cover the greatest possible number of CpG sites within the promoter region; necessary PCR amplification length, target sequence length, and primers that avoided CpGs were taken into account. DNA methylation levels at multiple CpG sites were measured and the mean methylation values for each gene were calculated. Significance was determined using a Student’s unpaired *t*-test using SAS (v9.1; SAS Institute Inc., Cary, NC, USA); a value of *P* < 0.05 was considered significant.

## Results

### Whole-genome BS-seq and analysis

To identify the dynamic changes in genomic methylation profiles at different growth stages, 324.73 Gb, 310.81 Gb, and 229.8 Gb of raw reads were generated for the mon1, mon24, and mon48 groups, respectively. After low quality reads (Ns > 10%, low-quality sites > 40%, adapter contamination, and duplication pairs) were removed from the data set, around 500 million clean reads were left for each group. In the three comparisons, 69.97%–84.48% of reads could be mapped (Table 2), and reads were mapped across the genome. The mapped reads were used for subsequent analyses.

**Table 2:** Summary statistics for whole-genome bisulfite sequencing data for lambs (1- and 24-month-old) and adult sheep (48-month-old) (mon1, mon24, and mon48, respectively)

Groups	Sample	Clean base, Gb	Clean reads	Mapped, %	Bisulfite conversion rate, %	Total mC, %
mon1	1–1	68.65	457,617,966	83.77	99.43	0.46
	1–2	80.78	538,546,500	77.94	99.6	0.05
	1–3	97.13	647,546,896	76.6	99.04	0.16
	1–4	78.17	521,158,720	73.45	99.26	1.6
mon24	24–1	66.27	441,789,090	81.51	99.52	0.19
	24–2	66.85	445,660,228	84.48	99.54	0.55
	24–3	83.62	557,475,654	77.41	99.1	0.19
	24–4	94.07	627,108,190	77.21	99.23	0.66
mon48	48–1	71.95	479,673,644	82.2	99.45	0.78
	48–2	82.97	553,128,086	69.97	99.56	2.24
	48–3	74.88	499,169,642	76.05	99.02	0.24

Methylation in sheep was found in three sequence contexts: CG, CHG, and CHH. These contexts were present in similar proportions in each group, with CG as the most abundant context by a wide margin; the methylation proportions of the mCG contexts were 97.69%, 97.22%, and 97.42% for mon1, mon24, and mon48, respectively. The proportions of the other mCHG and mCHH contexts ranged from 0.48%–0.52% and 1.82%–2.25%, respectively.

**Fig. 2:**
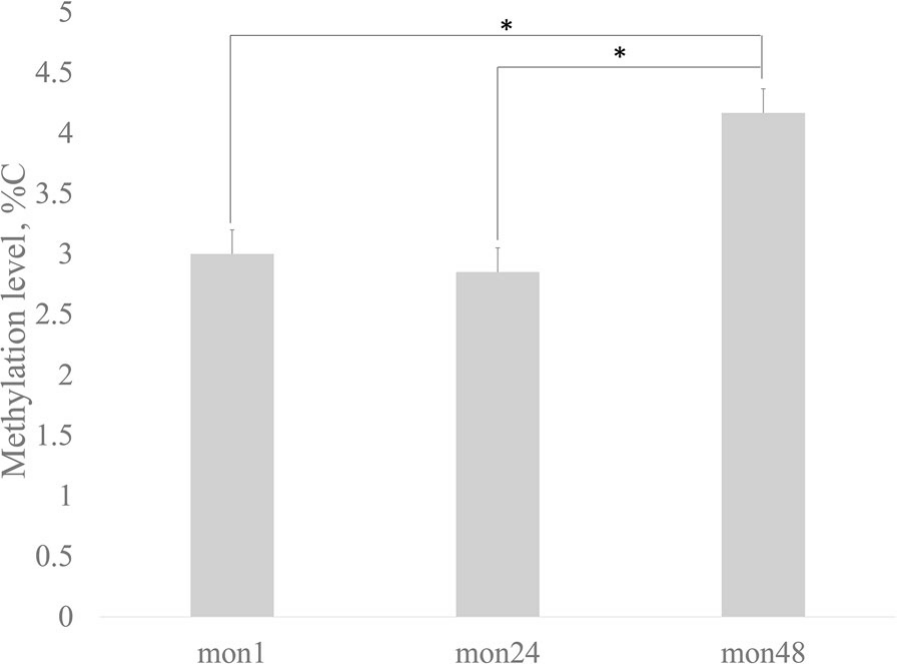
Distribution of DNA methylation levels in mon1, mon24, and mon48 groups (*P < 0.05)

Although methylation in different sequence contexts can be conveniently described using the statistics above, the frequency of DNA methylation varies by site, even within a single context. The frequency at a particular site can be estimated from the fraction of mapped reads that contain a methylcytosine residue at the appropriate position. This frequency was defined as the methylcytosine methylation level. The column diagram in Fig. 2 shows the distribution of mC levels for all methylated sites detected in each group. Although some sites were frequently methylated, the vast majority were methylated at relatively low levels.

**Fig. 3:**
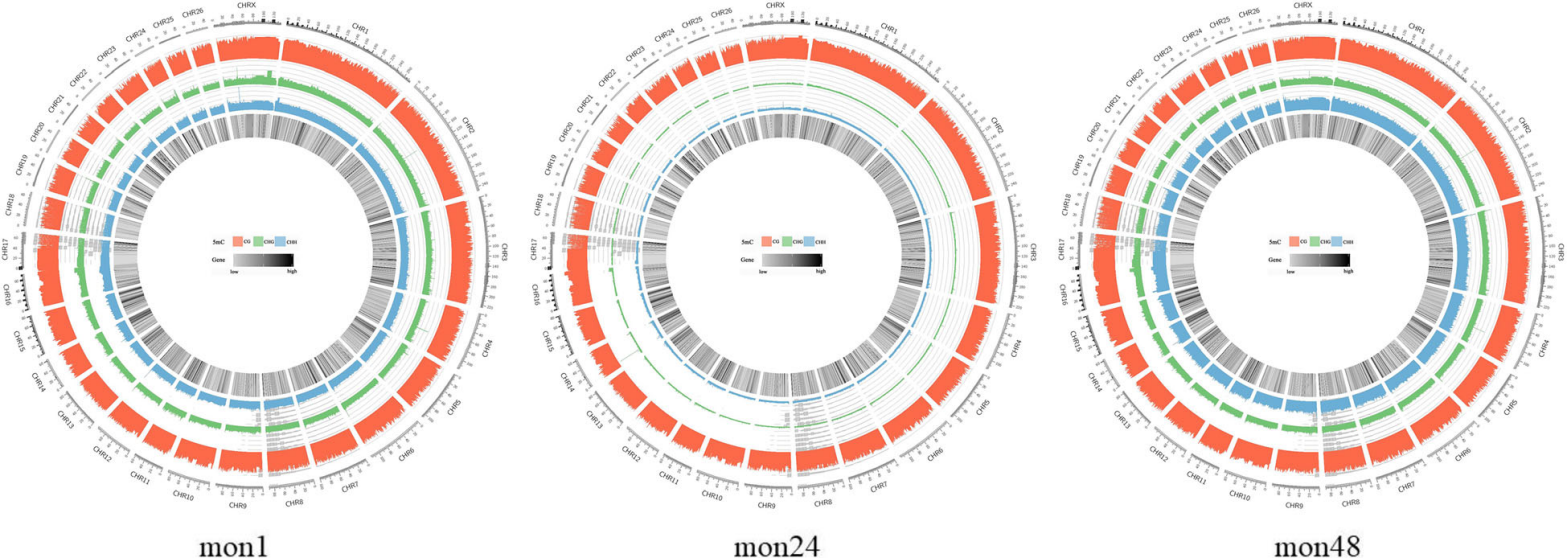
Genome-scale view of the Tan sheep shoulder skin methylome. The red, green, and blue bars represent the methylation levels in the CG, CHG and CHH context, respectively; highlighted gray bands refer to normal annotated genes

To visualize the distribution of methylated sites as a function of genome location, we used the graphical tool Circos [23] to generate large-scale methylation maps organized by chromosome (Fig. 3, Additional Fig. S1). Cytosine methylation levels in the CG (red), CHG (green), and CHH (blue) contexts are shown separately for mon1, mon24, and mon48 methylomes.

### Motif analysis of nearby mC at different contexts

To understand the base-pair distribution of mC, we analyzed the relationship between sequence context and methylation preference. The mC sites were in a hypermethylation state, the CAG was the most common sequence motif in the CHG mC sites, and similar frequencies were discovered in the CHH context for the three groups (Additional Fig. S1).

### Distribution of DNA methylation sites in functional genomic regions

**Fig. 4:**
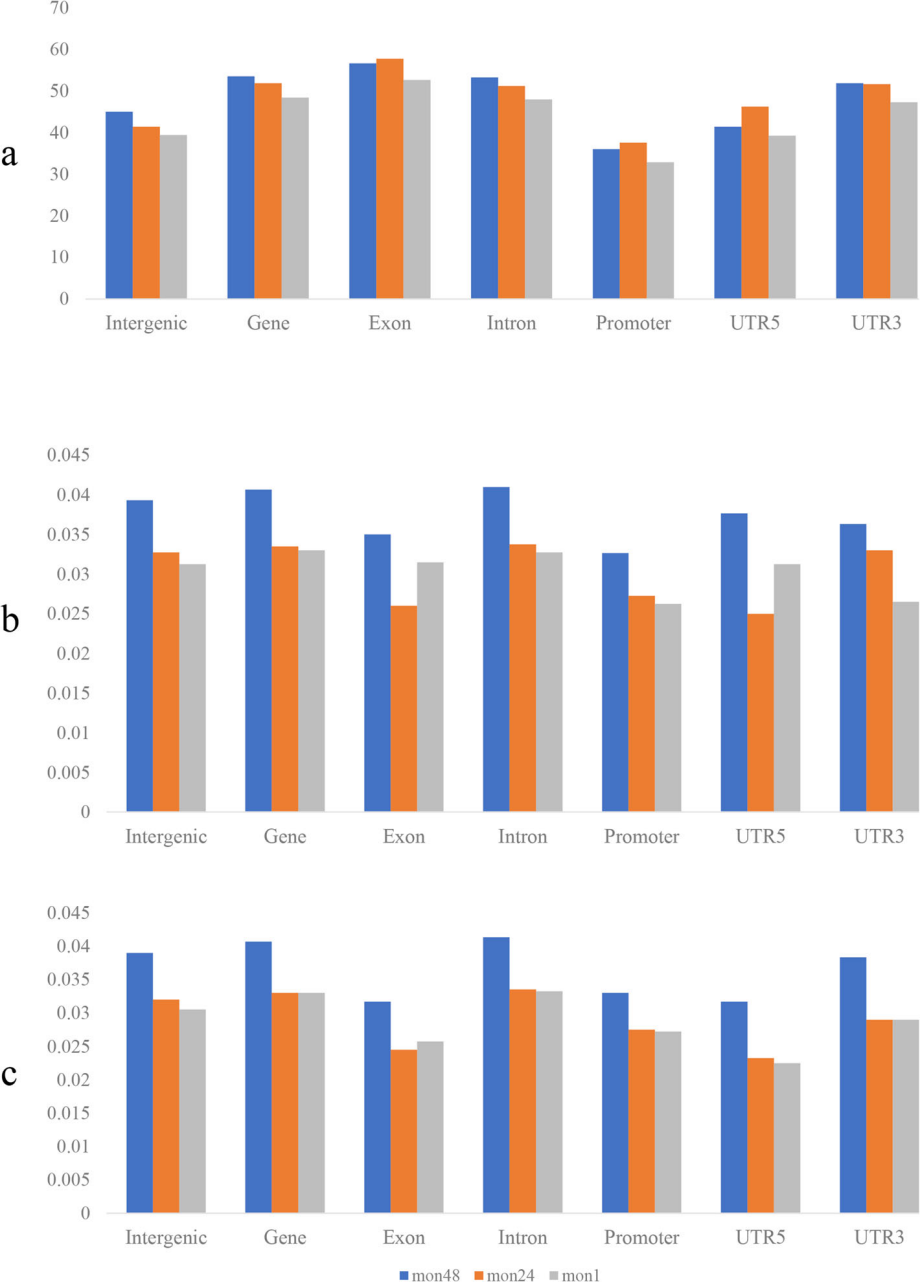
mC distribution in different functional elements. **a**, **b**, and **c** show the trends in mC distribution of different functional elements for CG, CHG, and CHH contexts, respectively. A, C, or T. The abscissa represents the different regions of gene functional elements (intergenic, gene, exon, intron, promoter, UTR5, and UTR3), and the ordinate represents the ratio of mC

Initial screening yielded 175,433 differentially methylated CpG sites between mon1 and mon24, 447,869 between mon1 and mon48, and 442,383 between mon24 and mon48. As shown in Fig. 4a, methylation levels for the CG context were similar in the three groups. Exon, UTR3, and gene regions contained the majority of mC sites. CG methylation levels in the promoter region were lower than those of the other elements; however, the levels showed a decreasing trend in intron regions. Methylation levels for the CHG and CHH contexts were higher in mon48 than the other two groups (Fig. 4b and c, respectively). These methylation levels were similar in mon1 and mon24, with lower methylation levels of the exon and UTR5 regions than other elements in the three groups.

### Identification of DMRs among tan sheep of three growth stages

**Fig. 5:**
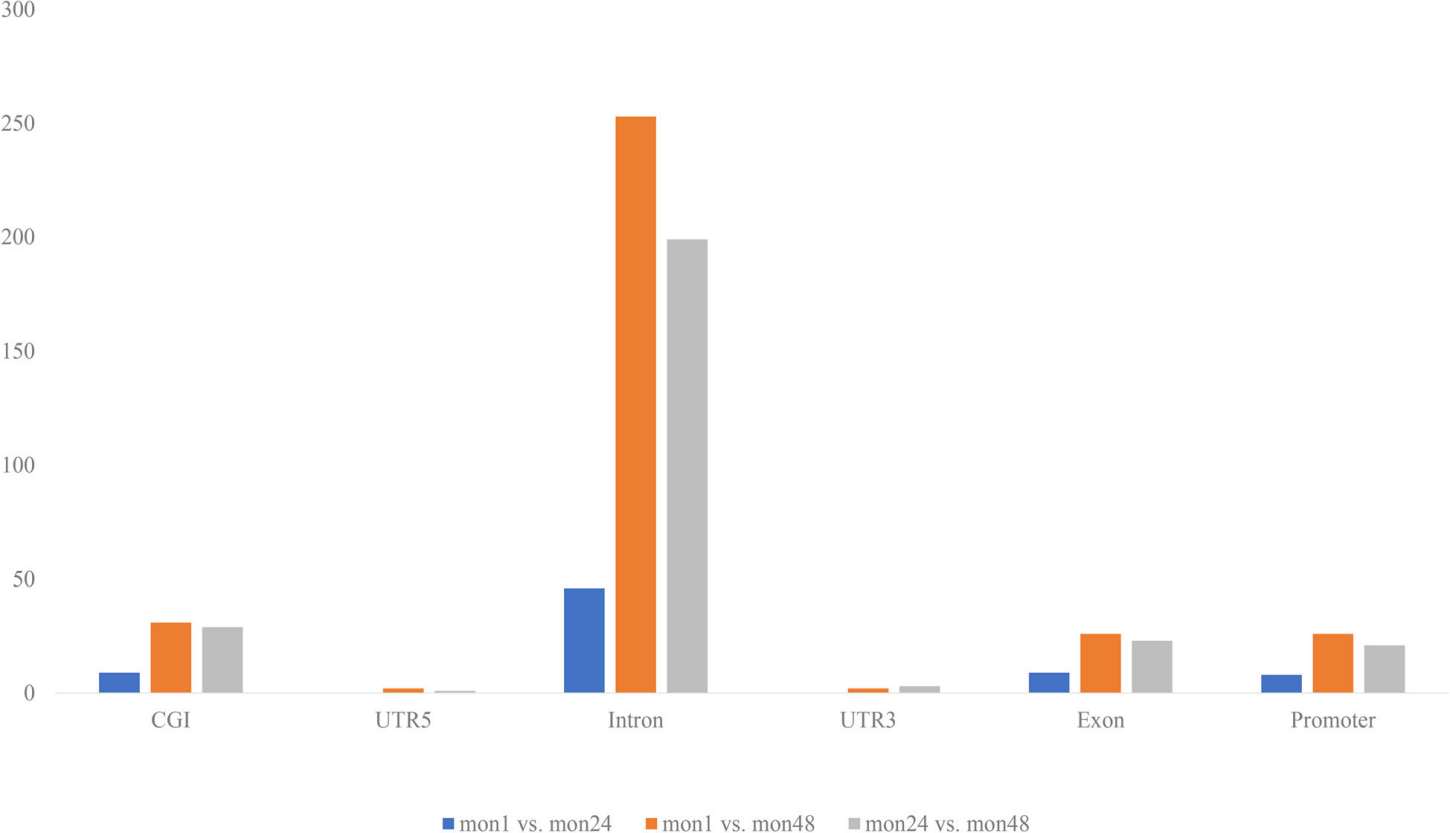
DMR distribution among gene functional elements

The analysis yielded 72 DMRs between mon1 and mon24, 340 DMRs between mon1 and mon48, and 276 DMRs between mon24 and mon48 (Additional file 1: Table S1). Most DMRs were in distal intergenic regions. Only 6, 25, and 20 DMRs were found in promoter regions between mon1 and mon24, mon1 and mon48, and mon24 and mon48, respectively. For DMRs that were in one of the seven described locations (intergenic, gene, exon, intron, promoter, UTR5, and UTR3), 63.89%, 72.1%, and 74.41% were in introns, and 1.11%, 7.61%, and 7.65% were in the promoter in mon1 vs. mon24, mon24 vs. mon48, and mon1 vs. mon48, respectively (Fig. 5).

**Fig. 6:**
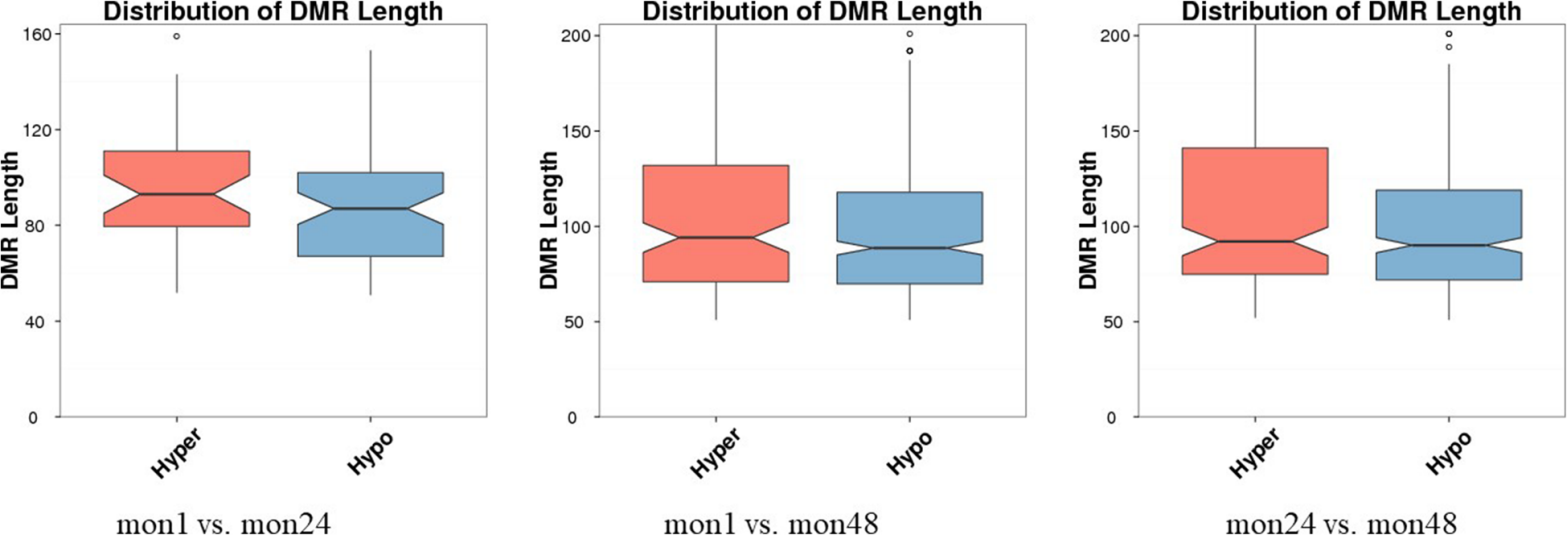
Length distribution of hypermethylated and hypomethylated DMRs (the methylation differences greater and less than 10%, respectively; *P* < 0.05)

The length distributions of hypermethylated (methylation differences greater than 10%, *P* < 0.05) and hypomethylated (methylation differences less than 10%, *P* < 0.05) DMRs of the three growth stages are shown in Fig. 6. The length distributions were very similar for all three comparisons. The results indicate that hypomethylated DMRs may be slightly shorter on average than hypermethylated DMRs. In total, the DMR length shortened with age, regardless of hypermethylation or hypomethylation status. In our data, the methylation levels of DMRs were influenced by age, and most showed high methylation in mon48. We inferred that the organismal metabolism slows and gene expression weakens with age. More detailed results are listed in Additional Table S1.

### Analyses of DMR-containing genes

GO analysis revealed that the CG type DMGs were significantly enriched in hair follicle morphogenesis (GO:0031069), hair follicle development (GO:0001942), and hair cycle processes (GO:0022405). KEGG analysis showed that the CG type DMGs were significantly enriched in the WNT (map 04310), MAPK (map 04010), and TGF-beta (map 04350) signaling pathways. Importantly, some DMGs were involved in biological processes important for cell differentiation (e.g., cell morphogenesis involved in differentiation, GO:0035120), which indicated that these genes may influence aging and fleece growth. For more detailed results from the GO and KEGG analyses of DMGs, see Additional Table S2, Additional Table S3 and Additional Fig. S2.

### Correlation analysis of DMGs and sheep growth

**Fig. 7:**
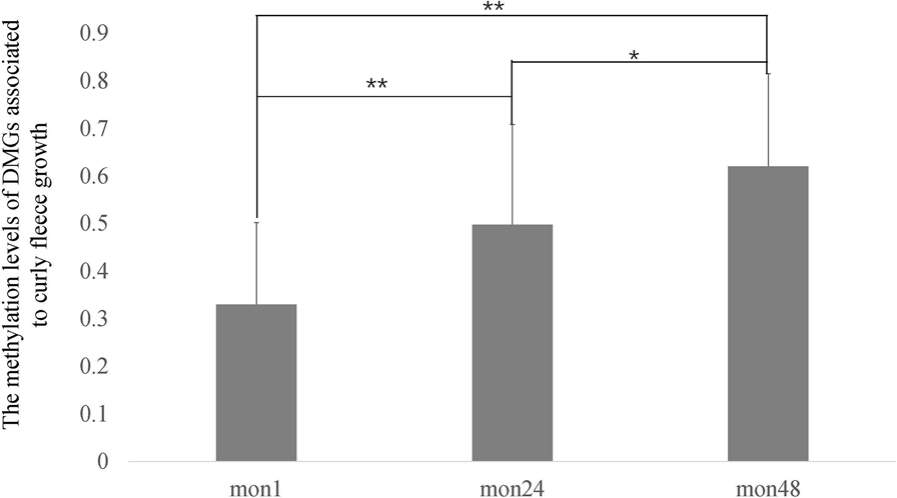
Methylation levels of DMGs related to curly fleece growth in mon1, mon24 and mon48. The ordinate represents the average methylation levels of DMGs, the abscissa represents the different growth stages (**P < 0.01; *P < 0.05)

To further understand the potential relationships between DNA methylation and growth stages, we performed an association analysis using two limiting factors: DMGs were enriched in fleece growth-related functions in GO analysis, and prior reports showed that these DMGs are related to growth. In our data, 51 genes were detected that met these two criteria (Table 3 and Additional Table S1). Some DMGs were hyper-methylated in mon1 that were hypomethylated in mon24 and mon48, and vice versa. Overall, methylation level showed a significant increasing trend with age in Chinese Tan sheep (*P* < 0.05) (Fig.7).

**Table 3:** Detailed information of DMGs associated with curly fleece formation

Chr	Direction	Group	Gene symbol	GO Term	KEGG
Chr1	Hypo	mon1	*PARP14*	GO:0005871	K15261
	Hyper	mon24	*CLDN8*	GO:0005198	K06087
	Hyper	mon48	*ATG4B*	GO:0004197	K08342
	Hyper	mon48	*VAV3*	GO:0005829	K05730
Chr10	Hypo	mon24	*NBEA*	GO:000588	
	Hyper	mon48	*ABCC4*	GO:0048661	K05673
	Hyper	mon48	*TNFRSF19*	GO:0001942	K05155
Chr12	Hyper	mon48	*TGFB2*	GO:0032147	K13376
Chr13	Hyper	mon24	*FRMD4A*	GO:0005856	
	Hypo	mon48	*STAM*	GO:0005070	K04705
	Hypo	mon48	*TAF4*	GO:1901796	K03129
Chr14	Hypo	mon1	*CKM*	GO:0005524	K00933
Chr16	Hyper	mon48	*CD44*	GO:0007156	K06805
Chr17	Hyper	mon24	*ARHGAP10*	GO:0007010	K13736
	Hyper	mon24	*NCOR2*	GO:0042826	K06065
	Hypo	mon48	*LRRC43*	GO:0007268	
	Hyper	mon48	*TBX3*	GO:0009887	K10177
Chr18	Hypo	mon48	*DLK1*	GO:0045746	
Chr2	Hypo	mon1	*SHB*	GO:0005070	
	Hypo	mon1	*RCC2*	GO:0090630	
	Hyper	mon24	*ASTN2*	GO:2000009	
	Hyper	mon48	*RCC2*	GO:0090630	
	Hyper	mon48	*SH3YL1*	GO:0035091	
	Hyper	mon48	*WNT10A*	GO:0005578	K01357
Chr20	Hypo	mon1	*GCLC*	GO:0007568	K11204
	Hyper	mon48	*GCLC*	GO:0007568	K11204
Chr21	Hyper	mon48	*PKP3*	GO:0002159	
Chr22	Hypo	mon48	*EMX2*	GO:0009952	K09317
	Hyper	mon48	*FGFR2*	GO:0001525	K05093
Chr23	Hyper	mon1	*MALT1*	GO:0007250	K07369
	Hyper	mon24	*DSG1*	GO:0016021	K07596
	Hyper	mon48	*NFATC1*	GO:0006351	
	Hyper	mon48	*SMAD2*	GO:0032924	K04500
Chr24	Hypo	mon1	*NSMCE1*	GO:0016874	
Chr25	Hyper	mon24	*ERCC6*	GO:0008023	K10841
	Hyper	mon48	*CDH23*	GO:0016021	K06813
Chr26	Hyper	mon24	*PDGFRL*	GO:0005576	
Chr3	Hypo	mon48	*MEIS1*	GO:0005667	K15613
Chr5	Hyper	mon24	*SMAD5*	GO:0030509	K16790
Chr6	Hypo	mon1	*LEF1*	GO:0008013	K04492
	Hyper	mon24	*FRAS1*	GO:0007154	
	Hyper	mon48	*CORIN*	GO:0004252	K09614
	Hyper	mon48	*EREG*	GO:0005154	K09784
Chr7	Hyper	mon48	*MCC*	GO:0090090	
Chr8	Hyper	mon48	*KRT71*	GO:0042787	K10468
Chr9	Hyper	mon48	*PRKDC*	GO:0006310	
ChrX	Hypo	mon1	*FGF13*	GO:0048487	K04358
	Hypo	mon48	*CLCN5*	GO:0031404	K05012
	Hypo	mon48	*COL4A5*	GO:0030574	K06237
	Hypo	mon48	*MBTPS2*	GO:0005737	K07765
	Hypo	mon48	*ZDHHC13*	GO:0005789	K16675

*Note*: Chr represents the chromosome; Hyper represents hypermethylation; Hypo represents hypomethylation

### DMR validation using BS-seq

**Fig. 8:**
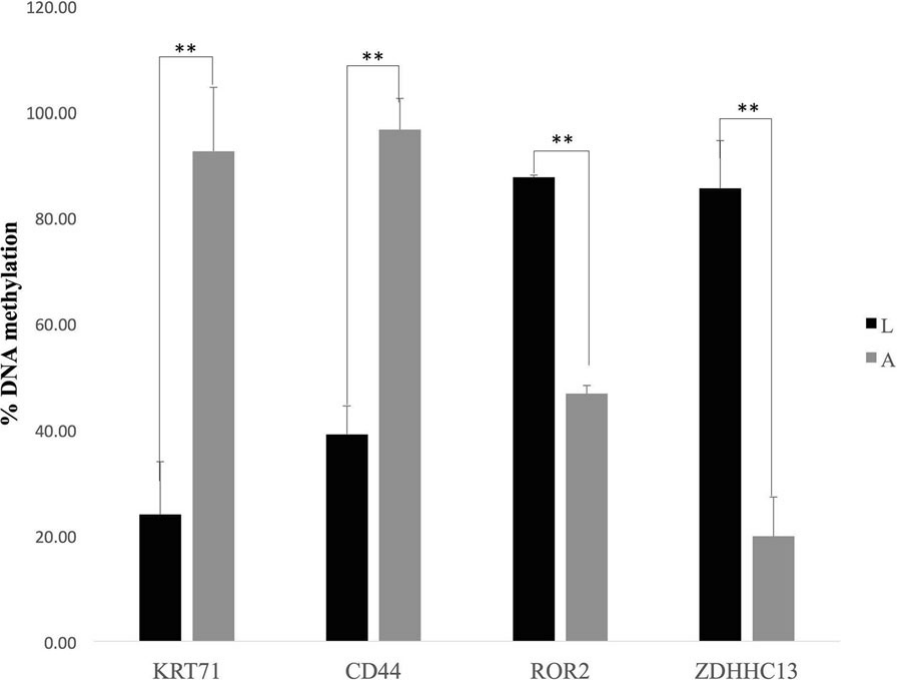
Methylation levels of four DMR-associated genes validated by pyrosequencing analysis between mon1 and mon48 (**P < 0.01)

The DMRs in *KRT71*, *CD44*, *ROR2*, and *ZDHHC13* identified by whole-genome DNA methylation analysis were selected for validation using the bisulfite Sanger sequencing method. The evaluated methylated CpG sites were all within the promoter region of these four genes. Methylation levels of the selected DMRs were consistent with the sequencing results (Fig. 8), BS-seq revealed that *KRT71* and *CD44* methylation levels were higher in mon48 than mon1 (*P* < 0.01), whereas *ROR2* and *ZDHHC13* exhibited higher methylation levels in mon1 than mon48 (*P* < 0.01). The concordance between these obtained data indicated that the DMGs identified by whole-genome BS-seq were credible.

## Discussion

Epigenetic modifications, such as changes in global and locus-specific DNA methylation, are suspected to play an important role in aging [24]. One possible mechanism is DNA methylation, which is involved in age-dependent animal phenotypes and age-related physiological variation [25]. A recent study revealed that DNA methylation patterns established in youth, in combination with other epigenetic marks, were able to accurately predict changes in transcript trajectories with aging [26]. In this study, methylation level showed a significant difference was observed between mon1 and mon48. Different methylation trends were found among Tan sheep at three growth stages, which confirmed the conclusion that the methylation patterns changed with age. The dynamic changes in DNA methylation of Tan sheep at different growth stages may represent regulatory mechanisms of changes in sheep biological development. DMGs play a major role in transcription regulation and are associated with either gene silencing or transcription elongation, depending upon the specific location of the methylated CpG sites in the gene [27]. In mammals, some transcription factors exhibit affinity to methylated DNA oligonucleotides outside their usual consensus motifs [28, 29]. In our study, 576 DMGs, which represented 1.42% of annotated genes within the current genome assembly, were identified between mon1 and mon24, mon1 and mon48, and mon24 and mon48. The ratio of DMGs in the promoter region between mon1 and mon48 was higher than that in the other two comparisons, which indicated that more genes silenced their function with age in Tan sheep. Therefore, these DMGs may be important post-selected genes for subsequent studies to address the relationship between methylation modifications and age in sheep.

GO and KEGG enrichment analyses revealed that the genes in DMRs were significantly enriched in the WNT, AMPK and MAPK signaling pathways; interestingly, most of these pathways are associated with wool development [30–33]. The WNT/β-catenin signaling pathway in particular plays an essential role in many aspects of development, and it is required for the initiation of curly hair development [34]. A previous study showed that diverse whole-genome methylation profiles characterized the two periods of HF growth (anagen and telogen), and increased DNA methylation may suppress HF development in anagen [35, 36]. IRF2 and STAT5A are transcription factors that serve as mediators that regulate transcriptional processes in HF growth and skin disease, and are affected by the methylation levels of binding genes [37]. We found 10 DMR-containing genes between mon1 and mon48 that were enriched in the WNT pathway, including *WNT10A*, *KRT71*, and *TXB3*, and some were already known to be involved in hair morphology. The functions of these DMRs require further validation in the future.

*WNT10A* was identified as a dermal papilla signature gene in mouse pelage follicles [38]. SNP analysis revealed that common variations in *WNT10A* have pleiotropic effects on hair morphology [39]. Moreover, other researchers found that *WNT10A* may participate in catagen by inhibiting keratinocyte proliferation and functioning as a WNT inhibitor [40]. In this study, we found that *WNT10A* was hypomethylated in mon1 but not mon24 or mon48. This result is consistent with the idea that *WNT10A* negatively affects curly hair growth [41]. *KRT71* is also of great importance in curly fleece growth. Several studies indicated that *KRT71* has positive effects on curly fleece growth [21, 42, 43]. Most previous studies modified the effects of *KRT71* by producing DNA sequence or transcription regulation variants, and our data showed that the methylation levels of *KRT71* changed in the three growth stages. Our BS-seq data showed that the methylation level of the *KRT71* promoter region increased with age. We speculate that methylation modification of *KRT71* is one reason for wool phenotype changes in Tan sheep. A previous study showed that *TBX3* was necessary in embryonic cells for specifying epithelial cell fates in organs that require epithelial-mesenchymal interactions for their development [38]. *TBX3* is another gene that is involved in the WNT pathway [44]. Our data showed that the methylation levels of *TBX3* differed in mon1, mon24, and mon48. However, the methylation levels showed a decreasing trend with age, which requires further investigation.

## Conclusions

This study reports the dynamic changes in genomic methylation profiles at different growth stages of Chinese Tan sheep. Differential DNA methylation profiles were observed in the three growth stages, and were as well associated with differential gene expression. We identified 51 DMGs related to age and fleece growth. Importantly, among DMGs, *KRT71, WNT10A*, and *TBX3* were identified in this study as potentially important genes associated with curly fleece formation, and previous studies demonstrated that these genes play significant roles in regulating keratinocyte proliferation and migration. The data presented here highlight the importance of epigenetic profiles in different growth stages of Tan sheep to help elucidate the variation of gene expression in different growth stages and improve the economic value in sheep.

## Supplementary Information

**Additional file 1 Table S1.** List of differentially methylated regions among three comparisons (mon1 vs. mon24; mon1 vs. mon48; mon24 vs. mon48).

**Additional file 2 Table S2.** GO analysis of DMR-associated genes.

**Additional file 3 Table S3.** KEGG pathway analysis for DMR-associated genes.

**Additional file 4 Fig. S1.** Motif of mCG sites in the genome. Methylation preferences in 9 bp spanning CG, CHG, and CHH methylcytosine sites. mon1 (mon1–1, mon1–2, mon1–3, mon1–4), curly fleece. mon24 (mon24–1, mon24–2, mon24–3, mon24–4), intermediate phenotype. mon48 (mon48–1, mon48–2, mon48–3), uncurled fleece. H = A, C, or T. The abscissa is the base number of the methylation site and the total height of each position is the sequence conservation of the base, which represents the relative frequency of the base at that position.

## Data Availability

Additional data can be found in supplementary files.

## Abbreviations

BS-Seq: Bisulfite sequencing
DMR: Differentially methylated region
FDR: False discovery rate
DMG: Differentially methylated gene
GO: Gene ontology
KEGG: Kyoto encyclopedia of genes and genomes
PCR: Polymerase chain reaction
mC: 5-methylated cytosines
HF: Hair follicle
