# Supplementary material for: Dynamic changes of genomic methylation profiles at different growth stages in Chinese Tan sheep
Source: J Anim Sci Biotechnol. 2021 Nov 2;12:118. doi: 10.1186/s40104-021-00632-9 (PMC8561971; doi:10.1186/s40104-021-00632-9)

Additional Fig.S1

mon1-1

high methylation low methylation medium methylation


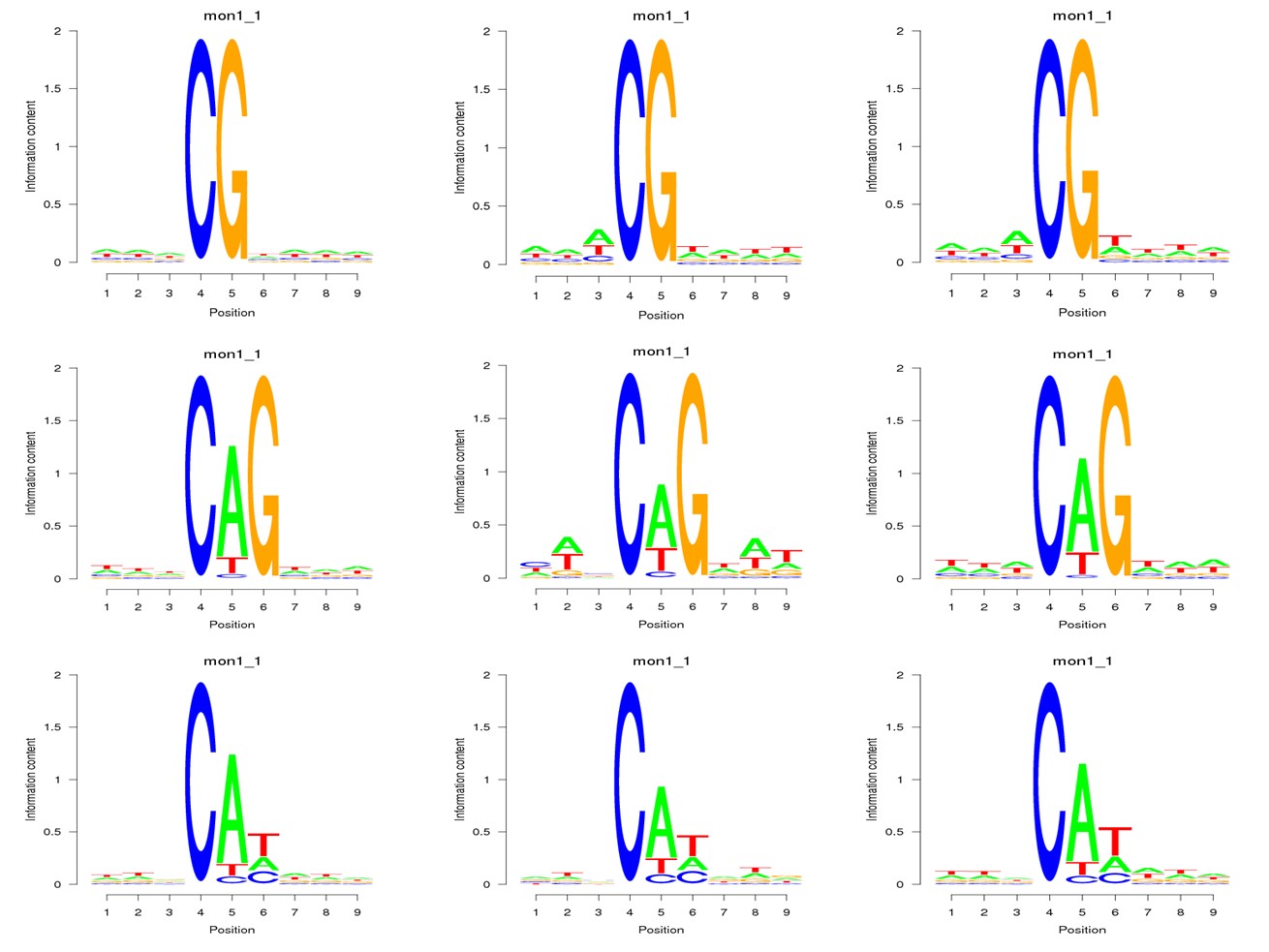


mon1-2


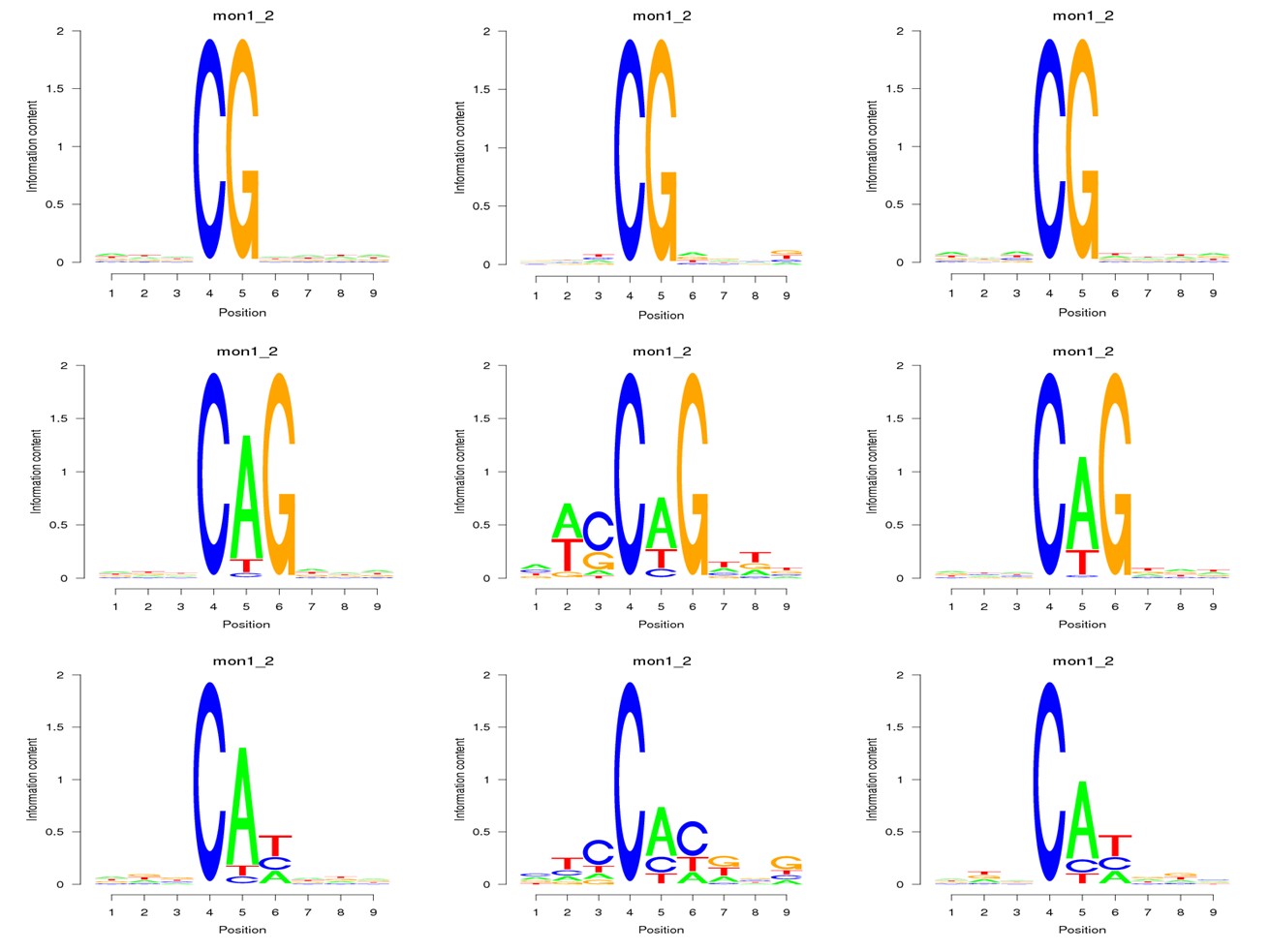


mon1-3


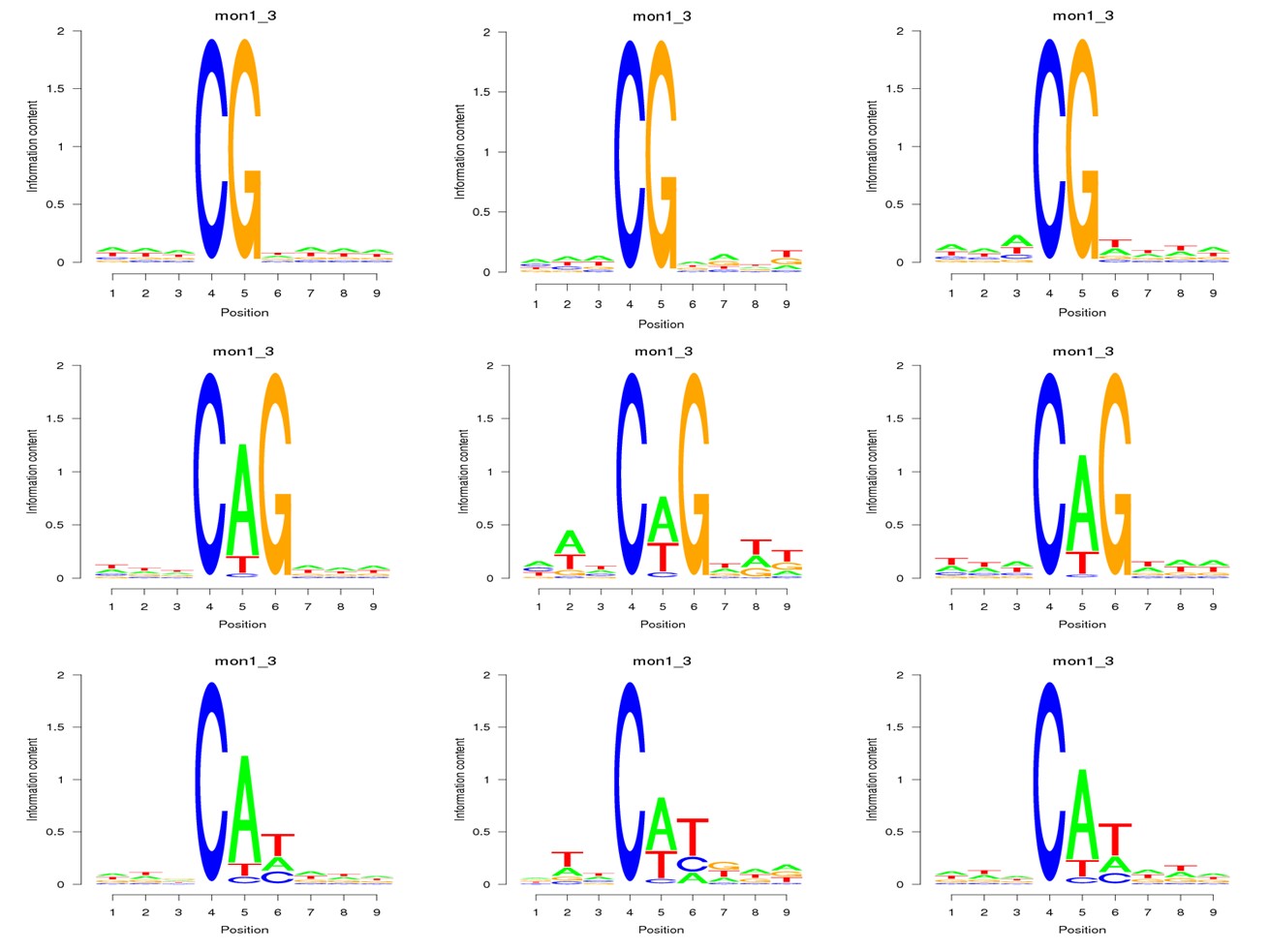


mon1-4


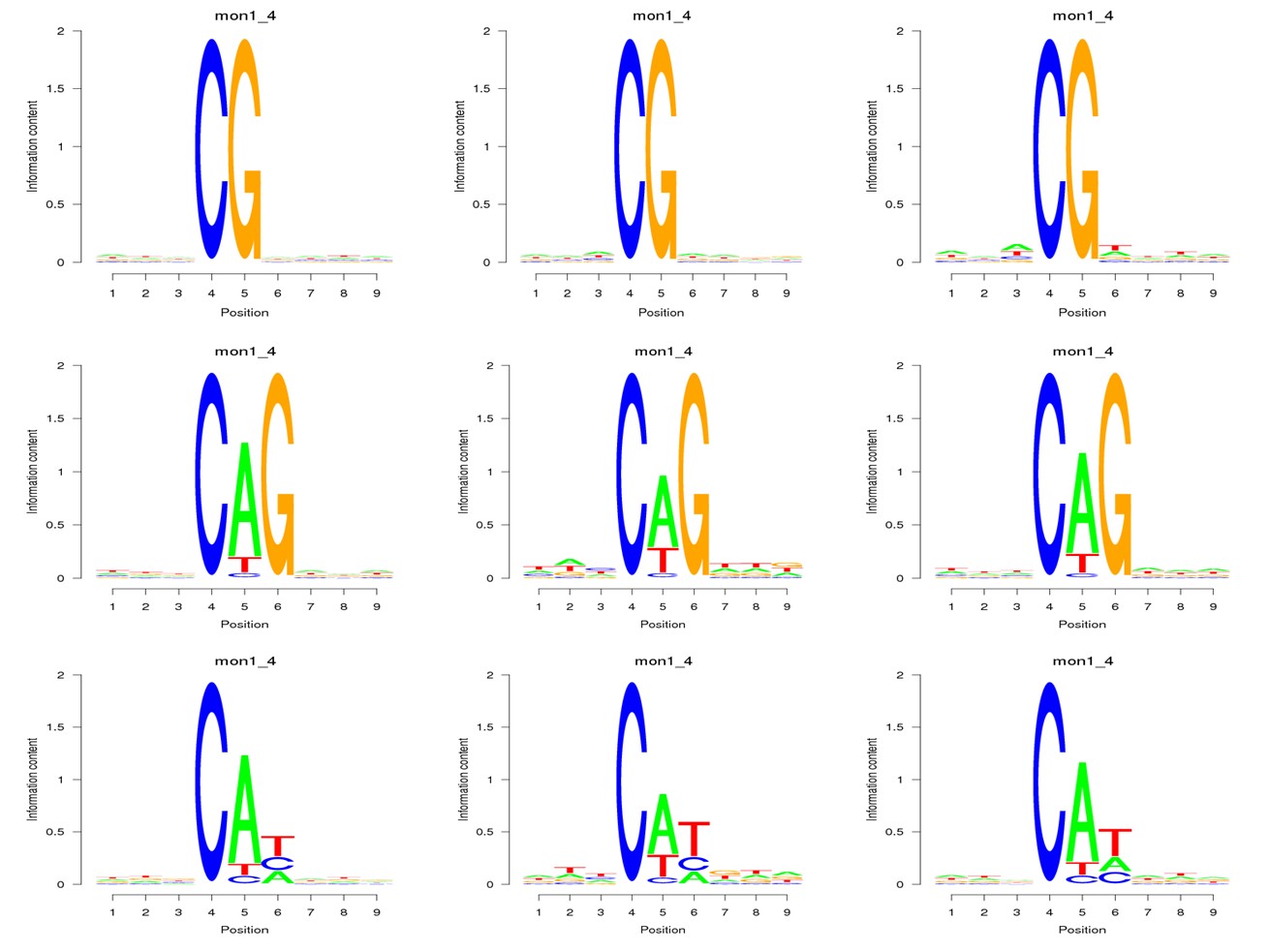


mon24-1


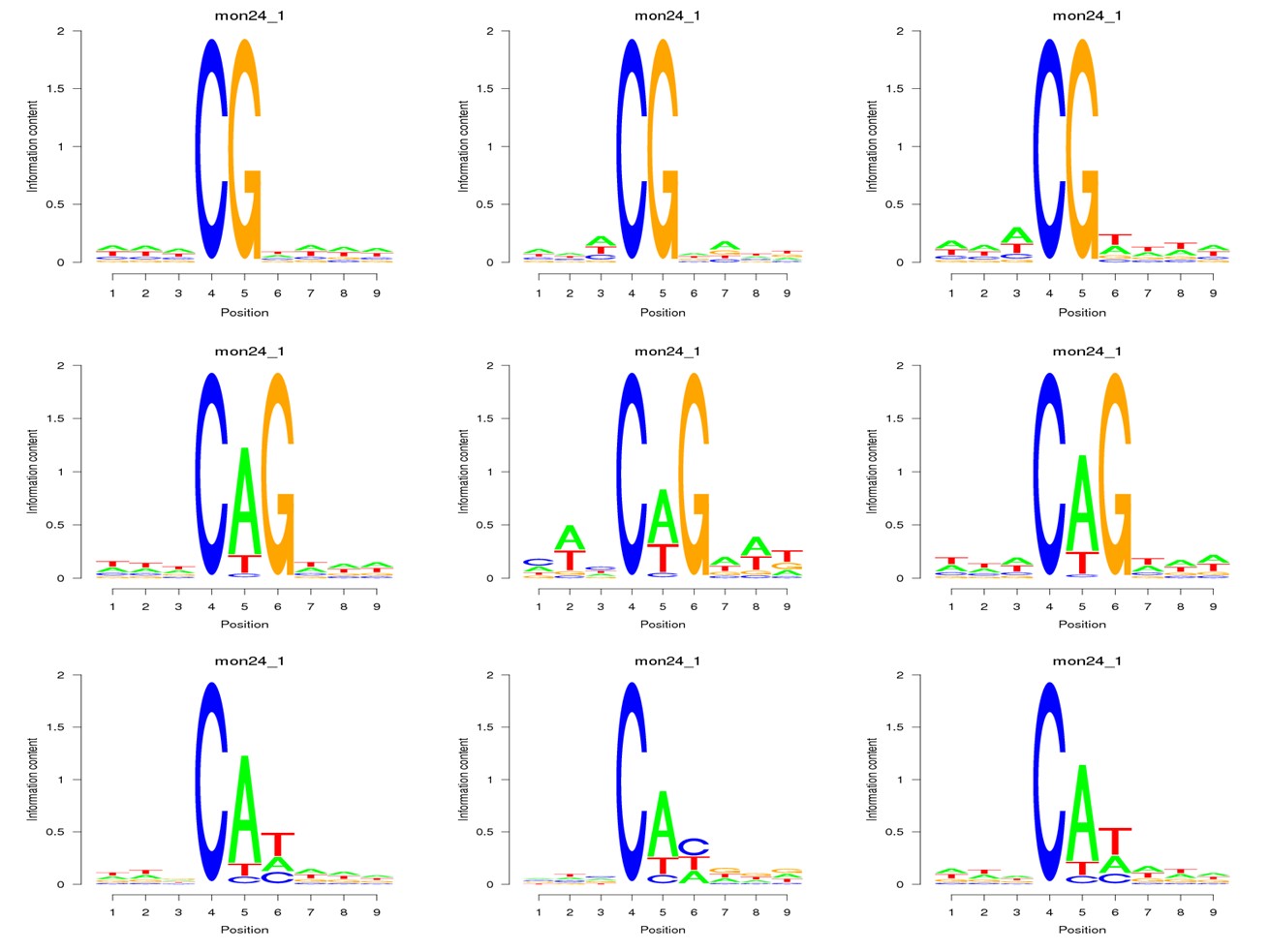


mon24-2


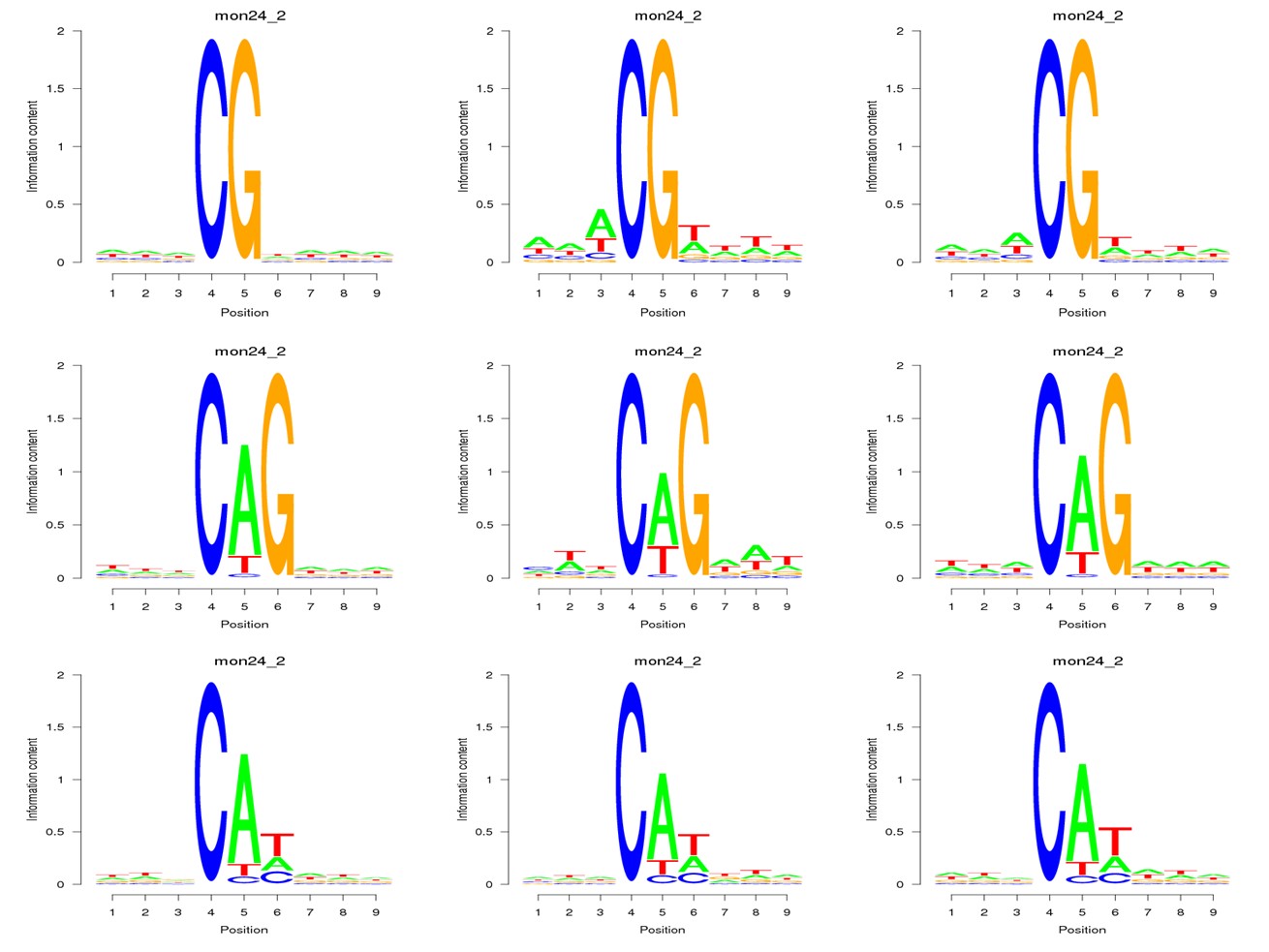


mon24-3


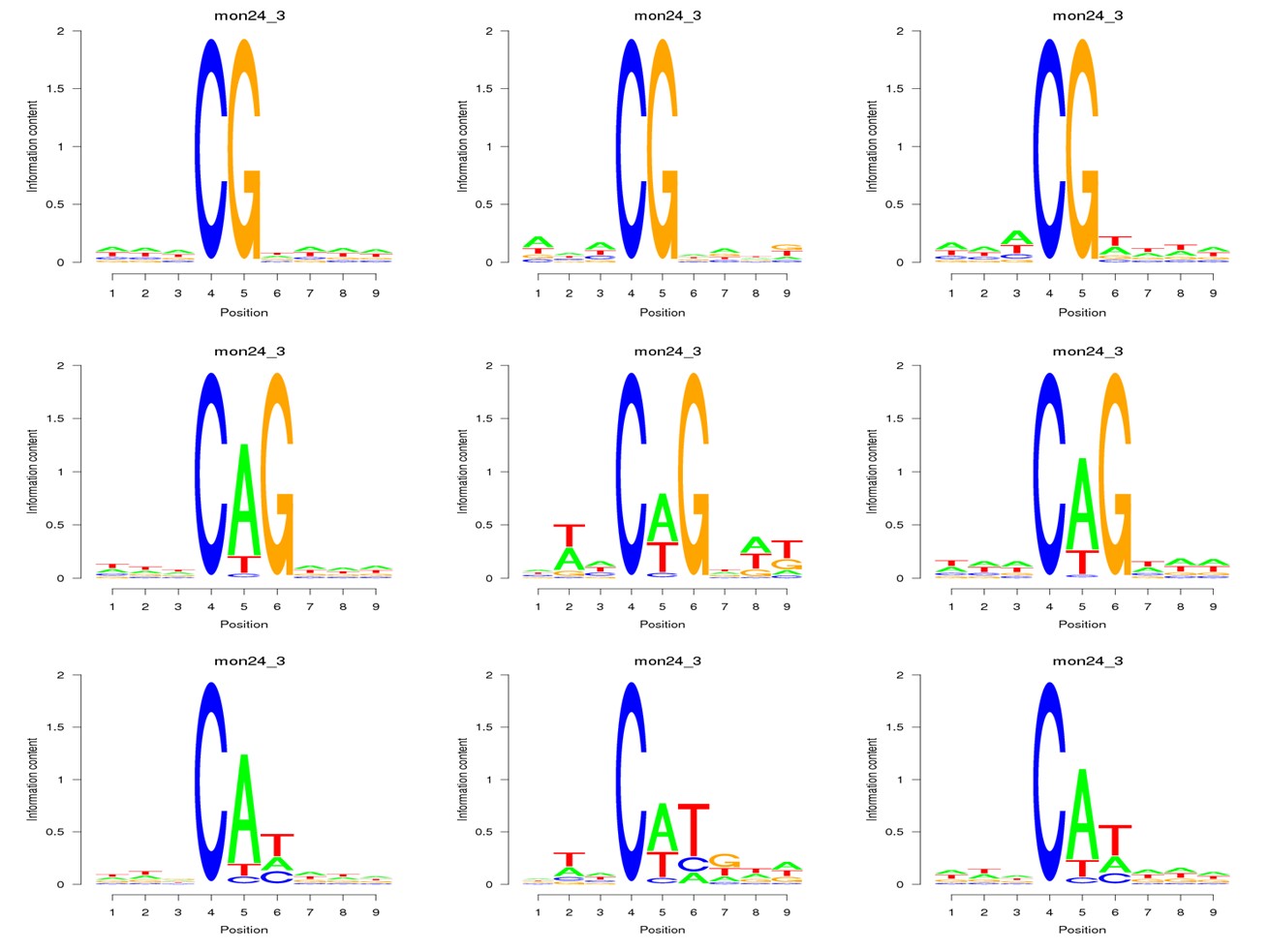


mon24-4


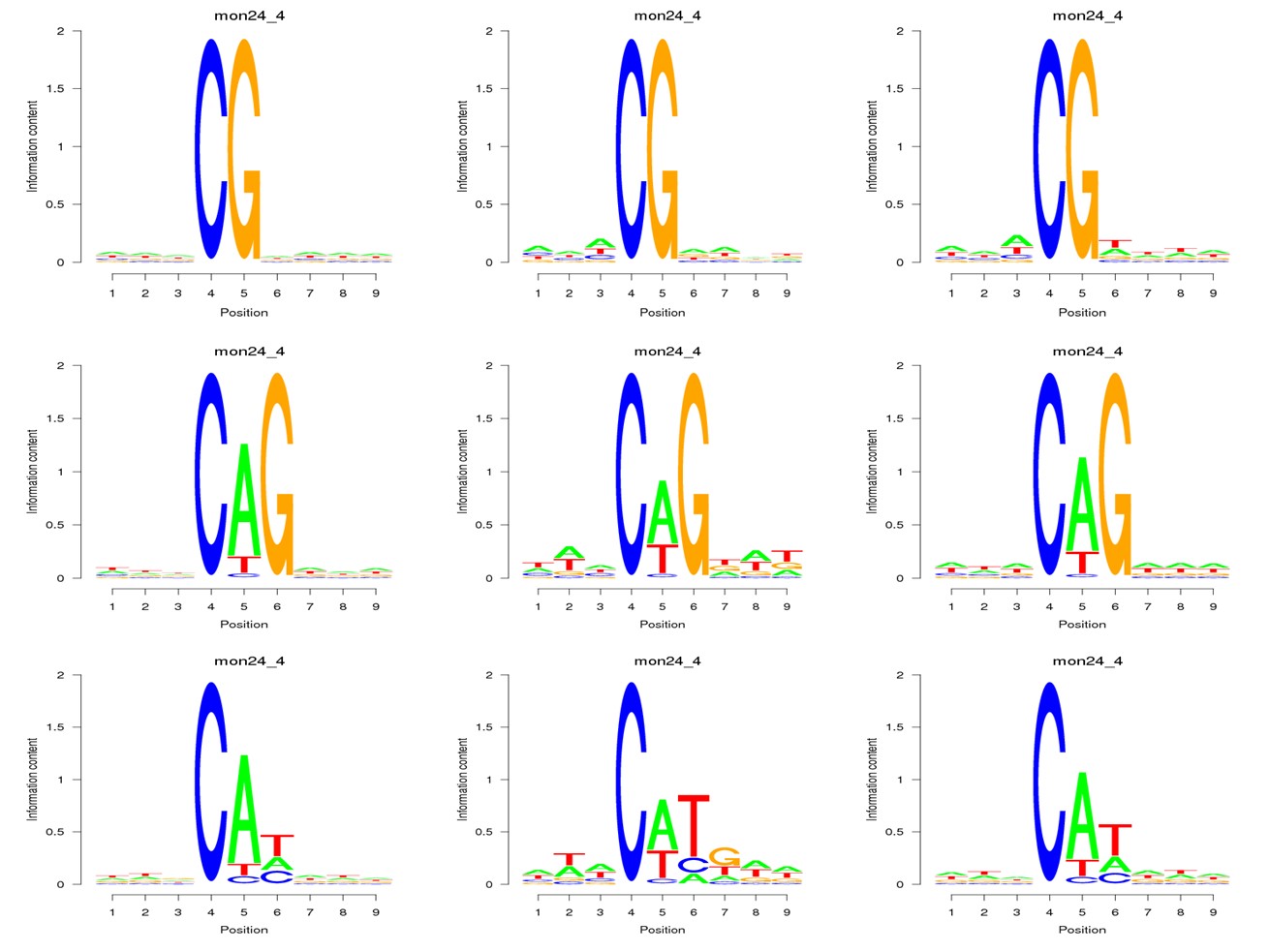


mon48-1


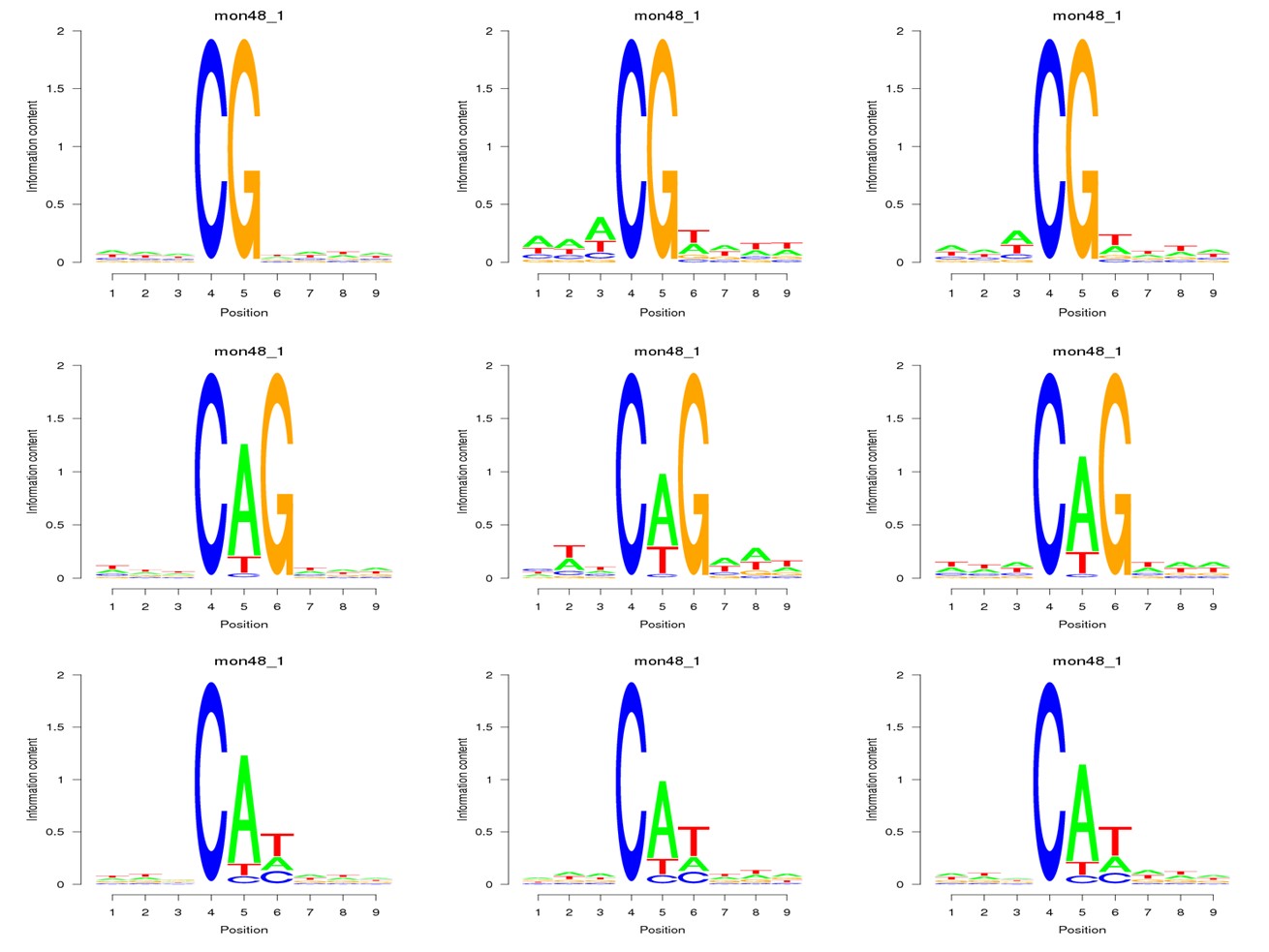


mon48-2


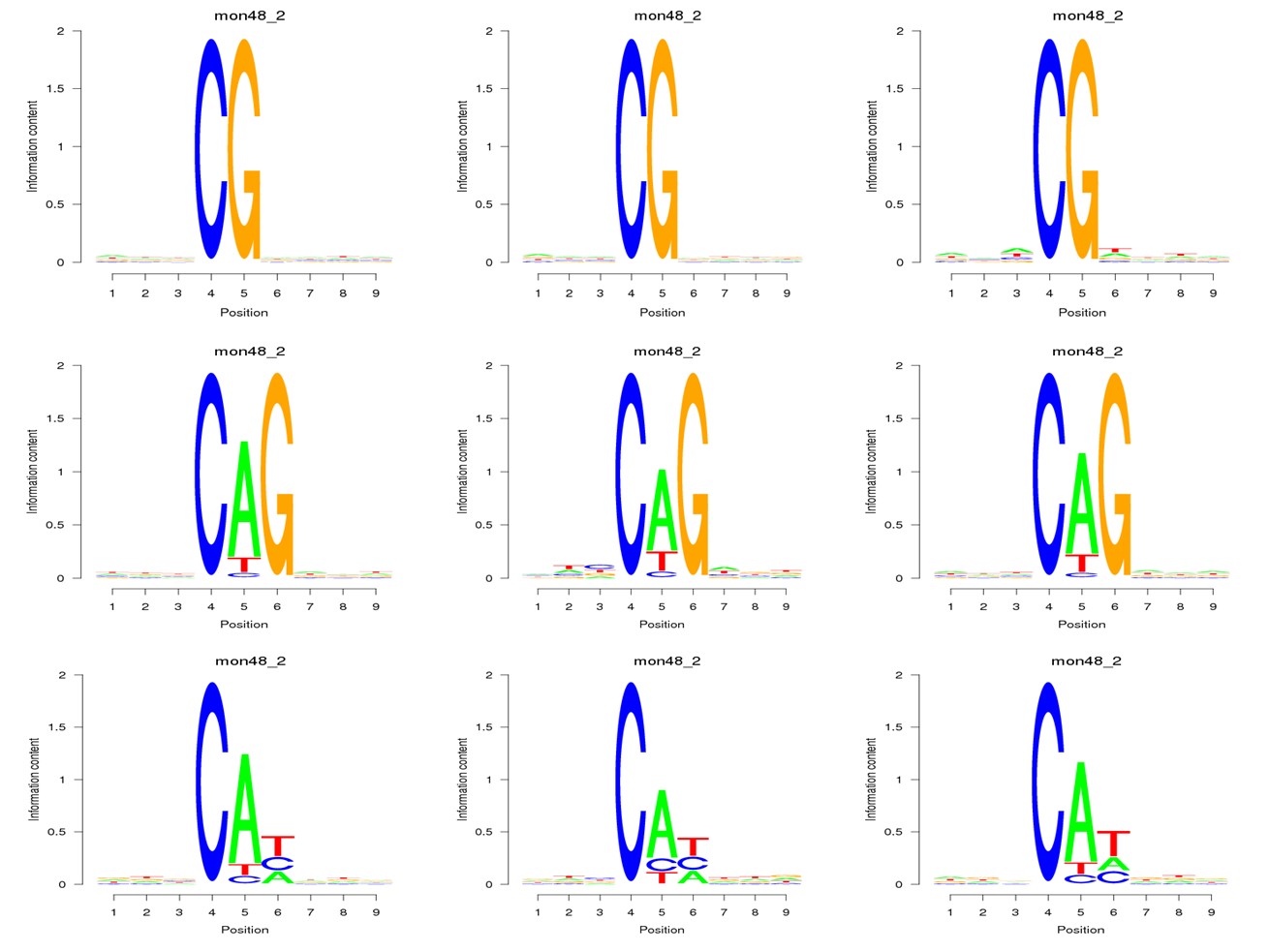


mon48-3


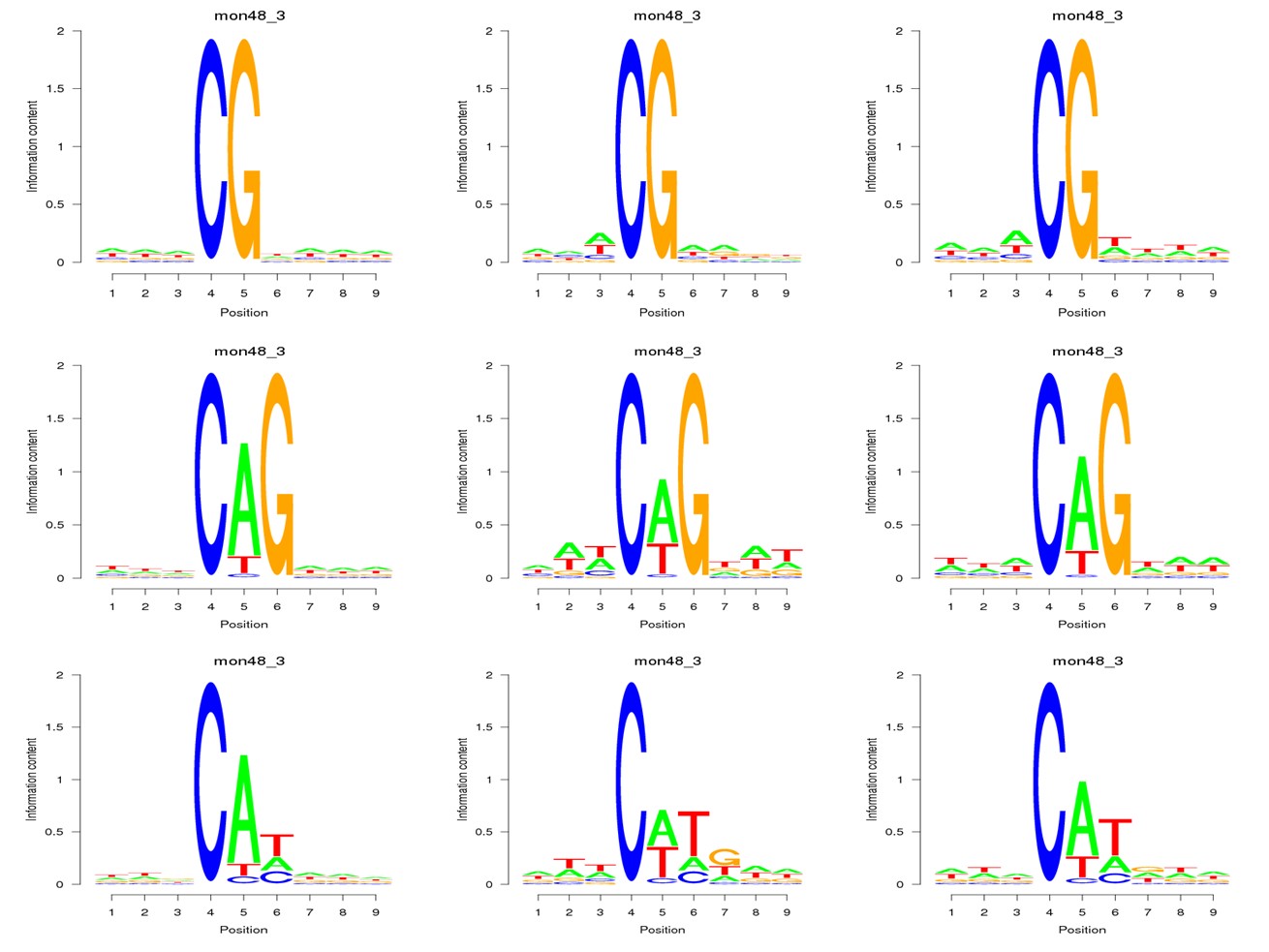

Supplement: Supplementary file 4 — Additional file 4 Fig. S1. Motif of mCG sites in the genome. Methylation preferences in 9 bp spanning CG, CHG, and CHH methylcytosine sites. mon1 (mon1–1, mon1–2, mon1–3, mon1–4), curly fleece. mon24 (mon24–1, mon24–2, mon24–3, mon24–4), intermediate phenotype. mon48 (mon48–1, mon48–2, mon48–3), uncurled fleece. H = A, C, or T. The abscissa is the base number of the methylation site and the total height of each position is the sequence conservation of the base, which represents the relative frequency of the base at that position. [file 40104_2021_632_MOESM4_ESM.docx]
